# The Present Position of Antiseptic Surgery

**Published:** 1891-04

**Authors:** 


### "THE PRESENT POSITION OF ANTISEPTIC SURGERY."

---

In August last, Sir Joseph Lister delivered an address at the Berlin International Congress on the above subject. A little later, that eccentric figure in modern surgery, Mr. Lawson Tait, published a criticism of the above address in the *British Medical Journal*, in which he denied the principles underlying the practice of antisepsis, and attacked the truth of the prevailing antiseptic methods.

His paper may be summed up in these words (his own): "Lister and his illogical disciples talk of the septic or antiseptic theory, where there is no theory about it at all, but an absolute and ludicrous logical error."

This attack has prompted Prof. White, of the University of Pennsylvania, to publish a defence of modern antisepsis (*Annals of Surgery*, January, 1891). We question if any defence were necessary. However, Prof. White presents facts and arguments which, we think, cannot be answered by the weak opposition. For example, in 1864, '65, '66, Prof. Lister's mortality in all operations was 45.7 per cent., largely from septic diseases. Now he begins his antiseptic methods, and in the next three years ('67, '68, '69) his mortality falls to 15 per cent. Still further improving his methods we find that from 1871 to 1877 in all surgical cases his mortality from septic disease was only 36 per cent. Again, compound fractures of leg and thigh under the old treatment had an average mortality of 40 to 50 per cent. After the introduction of antisepsis this fell to about 4 per cent.

Prof. White continues: Lister's work since he took his first prize in 1852 has been of a character to commend the respect and admiration of the scientific world. Prof. Louis Agassiz said years ago, and Prof. Joseph Leidy later, that his work had been of the highest order, and the appreciation of his labors by the best minds in our profession had been enthusiastic and almost universal. Nearly every great surgeon in the civilized world had put on record his admiration for Lister's teachings, his acceptance of the general principles involved, and his sense of almost personal obligation to the author of the antiseptic theory.

That Mr. Tait should speak of such a man as having "lived in the clouds of his spray for the last twelve years," as "wanting logic," making "illogical blunders," "falling away from his own faith," etc., and should boast of having "laughed at" and "ridiculed" him and his doctrines and disciples is evidence of his unfitness by temperament or training for the serious discussion of broad surgical principles. I am quite sure that the vast majority of general surgeons will be found to have no sympathy with his views or his manner of expressing them, and it is a relief to find that in his own special line there are operators of equal eminence who repudiate both.

